# Threshold for Onset of Injury in Chinook Salmon from Exposure to Impulsive Pile Driving Sounds

**DOI:** 10.1371/journal.pone.0038968

**Published:** 2012-06-20

**Authors:** Michele B. Halvorsen, Brandon M. Casper, Christa M. Woodley, Thomas J. Carlson, Arthur N. Popper

**Affiliations:** 1 Pacific Northwest National Laboratory, Marine Sciences Laboratory, Sequim, Washington, United States of America; 2 Department of Biology, University of Maryland, College Park, Maryland, United States of America; Institute of Marine Research, Norway

## Abstract

The risk of effects to fishes and other aquatic life from impulsive sound produced by activities such as pile driving and seismic exploration is increasing throughout the world, particularly with the increased exploitation of oceans for energy production. At the same time, there are few data that provide insight into the effects of these sounds on fishes. The goal of this study was to provide quantitative data to define the levels of impulsive sound that could result in the onset of barotrauma to fish. A High Intensity Controlled Impedance Fluid filled wave Tube was developed that enabled laboratory simulation of high-energy impulsive sound that were characteristic of aquatic far-field, plane-wave acoustic conditions. The sounds used were based upon the impulsive sounds generated by an impact hammer striking a steel shell pile. Neutrally buoyant juvenile Chinook salmon (*Oncorhynchus tshawytscha)* were exposed to impulsive sounds and subsequently evaluated for barotrauma injuries. Observed injuries ranged from mild hematomas at the lowest sound exposure levels to organ hemorrhage at the highest sound exposure levels. Frequency of observed injuries were used to compute a biological response weighted index (RWI) to evaluate the physiological impact of injuries at the different exposure levels. As single strike and cumulative sound exposure levels (SEL_ss_, SEL_cum_ respectively) increased, RWI values increased. Based on the results, tissue damage associated with adverse physiological costs occurred when the RWI was greater than 2. In terms of sound exposure levels a RWI of 2 was achieved for 1920 strikes by 177 dB re 1 µPa^2^⋅s SEL_ss_ yielding a SEL_cum_ of 210 dB re 1 µPa^2^⋅s, and for 960 strikes by 180 dB re 1 µPa^2^⋅s SEL_ss_ yielding a SEL_cum_ of 210 dB re 1 µPa^2^⋅s. These metrics define thresholds for onset of injury in juvenile Chinook salmon.

## Introduction

Over the past decade, there has been increased pressure for ocean exploitation in near shore and deeper waters for development of renewable energy and continued exploration for new energy resources. Pile driving and site exploration activities, such as seismic surveys, are often used in the construction of ocean wind- and hydrokinetic-farms, coastal bridges and docks, and liquid natural gas piers. These activities generate underwater impulsive sounds that have the potential to harm or kill fishes and/or result in behavioral changes that could adversely impact fish populations.

Despite the increase of impulsive sounds generated from the rise in pile driving and other activities, there is a lack of controlled experimental data concerning the effects from such exposures on fishes (e.g., [1], [2], [3], [4], [5], [6]). This is because it is difficult to implement rigorous experimental protocols required for biological exposure-response measurements during construction and exploration operations. In addition, the hazardous conditions and associated costs of construction operations prevent investigators from controlling ambient conditions and concomitant physiological conditions (e.g., buoyancy state) of test fish. Nor can investigators control the characteristics of impulsive sound exposure variables needed to understand and quantify the effects of impulsive sound on fishes [7], [8], [9].

**Figure 1:**
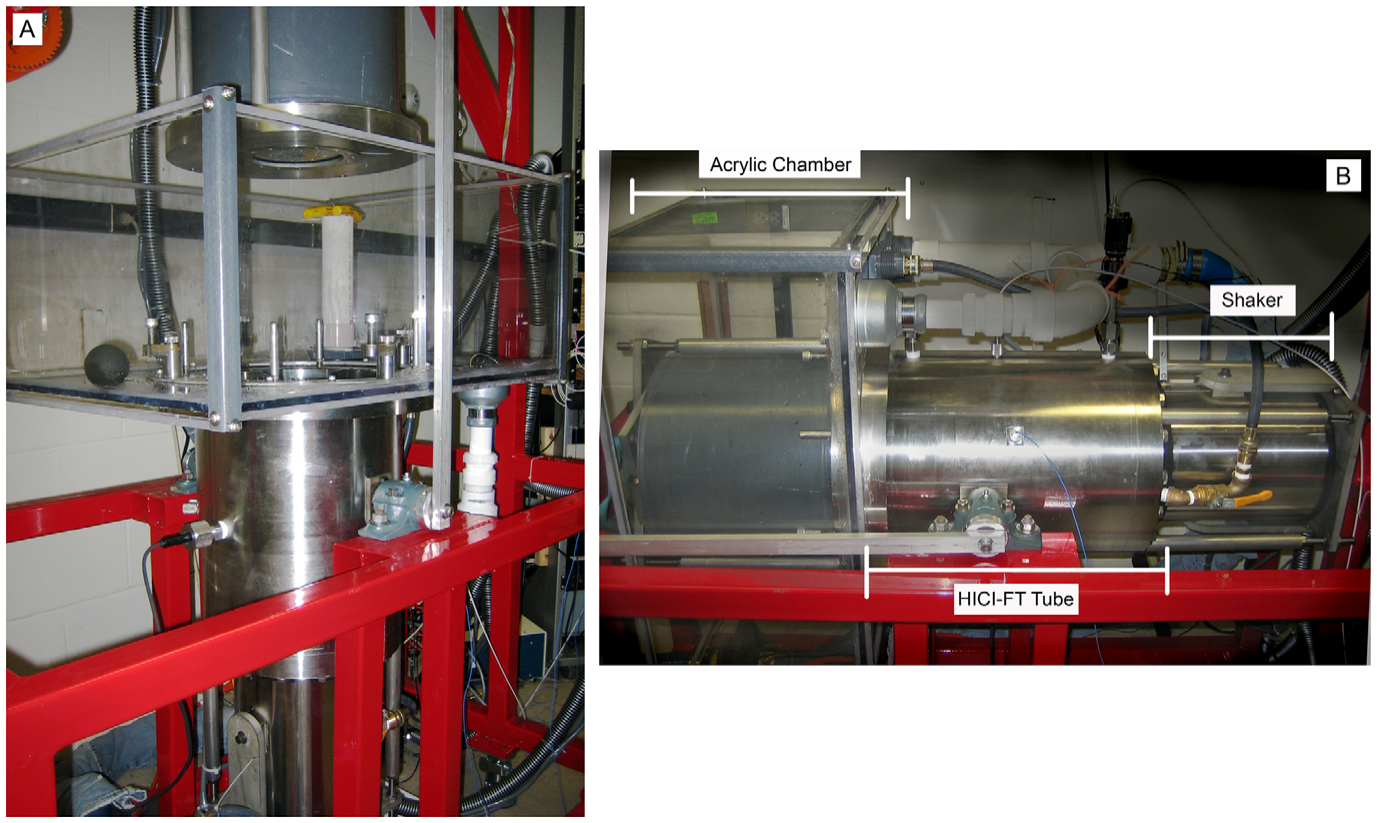
The HICI-FT in the horizontal and vertical positions. A) The HICI-FT in the vertical position for loading fish into the acrylic chamber. The top shaker is detached from the tube and is surrounded by gray PVC to protect it from the water in the acrylic chamber. B) During Treatments the HICI-FT is in the horizontal position. The shaker is labeled. The red structure is the supporting buggy, the white PVC pipes drain the water, and the grey hoses are part of the shaker cooling system.

Since well-controlled field studies on effects of impulsive sounds are generally not possible, a preferred alternative is laboratory-based studies [8], [10]. In the laboratory, investigators attempt to control environmental cues to minimize physiological responses unrelated to the desired exposure. Controlled impulsive sound exposures can also accurately simulate what the fish would experience under field conditions. This includes controlling variables such as the number of individual impulsive sound exposures, intervals between exposures, and single and cumulative impulsive sound exposure characteristics. Laboratory-based experiments also permit investigators to generate sounds over a wide range of intensities, thereby accurately representing those generated by actual construction operations. In the laboratory, it is difficult to reproduce such impulsive sound because they require the generation of very intense acoustic signals with properties representative of those in aquatic far-field, plane-wave conditions [8]. Plane-wave acoustic conditions are those that exist at distances greater than a few meters from any sound source such as pile driving or a survey vessel. Thus, to meet these challenges, this study required the development of a specially designed wave tube called a High Intensity Controlled Impedance – Fluid filled wave Tube (HICI-FT) [11], [12], see Figure 1. The HICI-FT provided the capacity to expose aquatic animals, such as fish and invertebrates, to impulsive sound under far-field, plane-wave acoustic conditions.

Barotrauma injuries from impulsive sounds primarily occur when there are instantaneous changes in volume and/or the state of gases in the body of fishes. Barotrauma is caused by rapid changes in gas volume and by rapid changes in the solubility of gas in the blood and tissues, such that as pressure increases solubility increases and vice versa. The rapid changes in gas states and pressures results in three categories of injuries: 1) emboli, 2) tissue damage caused by emboli, and 3) tissue damage caused by the expanding gas-filled organs. Emboli are created when gas leaves solution; it forms gas bubbles in the blood and body tissues (i.e. decompression sickness). The presence of emboli increase vessel pressures and can cause vessels to rupture and/or can disrupt the function or damage vital organs such as the heart, kidney, and brain [13], [14], [15].

Changes in volume occur when free gas in the swim bladder or in naturally-occurring bubbles in the blood and tissues expand and contract during rapid pressure changes, leading to tissue damage. Expanding gas-filled bodies (i.e., swim bladder) push against surrounding tissues at a high magnitude and rate of volume change, thereby damaging surrounding tissues over the duration of the motion [8], [9], [14], [15]. In some cases, the swim bladder itself could rupture, leading to disruption of swimming performance and buoyancy control. Negatively buoyant fishes are substantially less prone to barotrauma injury because the lack of buoyancy prior to exposure decreases the pressure effects on the swim bladder and internal gasses, thereby protecting the fish from barotrauma [9]. The most severe effects, such as bubbles in the gills or heart, may result in immediate death at exposure from impulsive sounds. Even if an injury is not immediately mortal, there may be delayed mortality resulting from injury processes such as hemorrhaging or indirect mortality from predation.

The goal for this study was to evaluate the effects, in terms of injury to tissues, for neutrally buoyant juvenile Chinook salmon (*Oncorhynchus tshawytscha*) exposed to high-intensity, impulsive sound. Specific objectives were to identify the exposure threshold at which onset of physical injury occurred, and to quantitatively describe the response of test fish to increases in the severity of exposure in terms of both the energy in the individual impulsive sounds contained in a Treatment and the total energy in all of the impulsive sounds comprising a Treatment.

## Materials and Methods

### Ethics Statement

Experiments were conducted under supervision and approval of the Institutional Animal Care and Use Committee (IACUC) of the University of Maryland (protocol #R-07-49).

### Study Fish

This study used juvenile Chinook salmon provided by the Pacific Northwest National Laboratory from the Priest Rapids Hatchery in Mattawa, Washington. Test fish had an average standard length of 103 mm ±8.75 (SD) and an average weight of 11.8 g ±3.47. Fish were held under the authority of the Maryland Department of Natural Resources (Natural Resources Articles 4-602 and 4-11A-02). A detailed description of the methods, including specifics on design of the HICI-FT and the approach to necropsy are presented in Halvorsen et al. [8].

### Preparation of Fish for Exposure

The physiological condition of fishes which includes their buoyancy state is an important biological issue for experiments in the field or in the laboratory. To avoid introduction of poor condition fish into this study, fish showing signs of stress or illness were not used. Previous studies on the effects of rapid changes in pressure on fish tissues have determined that the buoyancy state of test fish at exposure is a factor in their response [9]. Physostomous fishes, such as salmonids, need to gulp or expel air to achieve neutral buoyancy. Fish selected for testing were placed into the HICI-FT’s acrylic chamber (Figure 1) and allowed 20 min. to have the opportunity to gulp air. After 20 min., each fish’s buoyancy status was visually determined as being negative, neutral, or positive. Fish entered the HICI-FT tube, which was then closed by locking down the top shaker. Fish determined not to be neutrally buoyant continued through the Treatment (exposure or control) to maintain protocol consistency, but were removed from study analysis. Control fish underwent the same process as sound-exposed fish, but without the impulsive sound exposure.

**Figure 2:**
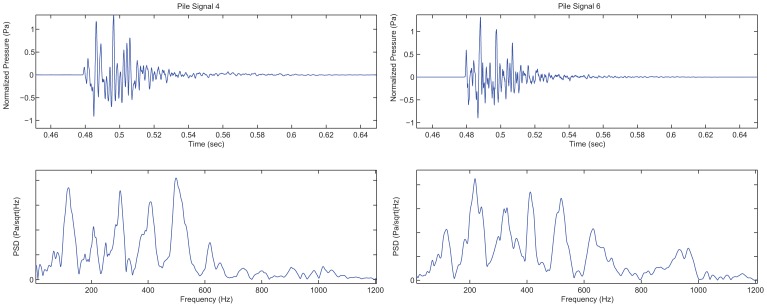
Two of the pile driving sound signals used in the study. In each figure pair, the upper image shows the signal time-domain normalized in amplitude, while the lower panel shows the power spectral density.

### Sound Exposure Methods and the HICI-FT

Sound exposure was conducted in the HICI-FT. The HICI-FT tube was a cylinder 0.45 m long with a 0.25 m internal diameter and 3.81 cm thick stainless steel walls. Fixed at each end of the tube was a shaker (Vibration Test Systems, VG-150 Vibration Generator, Model VTS 150, Aurora, OH) with a rigid face-plate mounted on the piston driven by a moving coil (Figure 1). The shakers were controlled independently in amplitude and phase to generate plane-wave pressure and velocity fields within the tube.

The HICI-FT produced highly accurate simulation of impulsive sound generated by pile driving. It could produce impulsive sounds with propagating plane wave characteristics up to peak sound pressure levels (SPL) of 215 dB re 1 µPa. The overall system permitted control of all exposure variables including the number of impulsive sounds and the details of individual impulsive sounds such as their duration, amplitude characteristics, and their energy. Control of these factors enabled precise control of the total energy in an exposure event.

Sound presentation was controlled using LabVIEW (National Instruments Corporation, Austin, Texas). Impulsive sound levels in the tube were continuously monitored and recorded using a hydrophone mounted inside the HICI-FT (Brüel & Kjær Sound & Vibration Measurement A/S, Naerum, Denmark, Model 8103). Throughout each Treatment, sounds were captured by the hydrophone, digitized, and stored.

### Sounds

Many different metrics are used in reporting underwater sounds to help define the acoustic characteristics of a signal. Often metrics are correlated or related to other metrics, for example, peak sound pressure level (SPLpeak) and single impulse sound exposure level (SELss) are highly correlated for impulsive pile driving impact sounds (Carlson et al., 2007b). This study used sound exposure level (SEL) and cumulative sound exposure level (SELcum) as independent variables. SEL is defined as the log transformed integral of squared sound pressure over the duration of a single sound impulse in dB referenced to 1 µPa2⋅s (Equation 1; ANSI S1.1). SELcum is defined as the sound exposure level over a number of individual impulsive sound exposures and is calculated as the log transformed sum of the squared sound pressure of the individual events (Equation 2). In practice, the sum of squared pressures are calculated for the portion of the impulsive signal containing 90% of the energy of the impulse. The impulsive pile driving sounds were normalized to have equal SELss value, so the computation of SELcum could be simplified (shown in Equation 2) as the sum of SELss and the log transform of the number of impulsive pile strikes in an exposure in dB referenced to 1 µPa2⋅s.



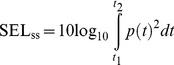
(1)

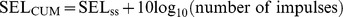
(2)


Eight impulsive sounds with different amplitude characteristics (examples shown in Figure 2) were used in this study. These sounds were analogues of field observations of both the pressure and particle motion components of impulsive sounds recorded at a range of 10 m from a diesel hammer that was driving a steel shell pile at the Eagle Harbor Maintenance Facility [16]. The impulsive sound exposure paradigms were designed to mimic the characteristics of pile driving sound impulses, the time between individual impulses, and the number of impulses of which 1920 and 960 present an average number of strikes needed to drive single piles. Furthermore, these two values were integral for testing the equal energy hypothesis.

The eight impulsive sound signals were normalized to the same SEL_ss_ and compiled into a file that contained 12 repetitions of each of the eight sounds for a total of 96 pile strikes in one file. In each repetition within a file, the location of each sound was randomized, and a new randomization was used on each study day. In each Treatment, the randomized file of 96 impulsive sounds was repeated 10 times for a 960 impulsive sound presentation or 20 times for a 1920 impulsive sound presentation. The duration of exposure was 24 minutes for 960 impulsive sounds, and 48 minutes for 1920 impulsive sound exposure.

### General Experimental Procedures

Fish were exposed to one of eleven impulsive sound Treatments that varied in total energy - SEL_cum_, single impulse - SEL_ss_, and in number of impulsive sounds (Table 1). Except for Treatment 1 (see below for explanation), all Treatments were conducted in pairs to achieve the same SEL_cum_ value with either 1920 or 960 impulses. The Treatment pairs differed in the energy per impulsive sound, SEL_ss_. To achieve the same SEL_cum_ value, the Treatment with 960 impulsive sound exposures needed a higher SEL_ss_ value (concomitantly higher SPL_peak_) than the Treatment for 1920 impulsive sound exposures. The maximum output level that could be generated by the HICI-FT was SPL_peak_ of 215 dB re 1 µPa. A Treatment producing 220 dB SEL_cum_ over 960 impulsive sound exposures was not achievable with the HICI-FT because of the sound pressure levels required to meet the SEL_ss_ requirement for this exposure condition. Therefore, the Treatment 1 exposure at 220 dB SEL_cum_ could only be conducted for the 1920 impulsive sound exposures.

**Table 1:** Exposure Treatments listed in order of SEL_cum_ and Number of strikes.

Treatment No.	Avg. SEL_cum_	Number of Strikes	Avg. SEL_ss_	Avg. SPL_Peak_	Duration, min	ExposedFish, n	ControlFish, n	Avg. RWI
1	220	1920	187	213	48	44	33	15.34
3	216	960	186	213	24	28	10	6.07
2	216	1920	183	210	48	36	16	5.97
5	213	960	183	210	24	31	7	4.32
4	213	1920	180	207	48	26	5	2.35
8	210	960	180	208	24	31	10	4.03
9	210	1920	177	204	48	30	11	3.43
6	207	960	177	203	24	24	8	1.04
7	207	1920	174	201	48	43	17	0.58
10	204	960	174	201	24	32	11	0.66
11	204	1920	171	199	48	31	12	0.42

### Barotrauma Assessment

**Figure 3:**
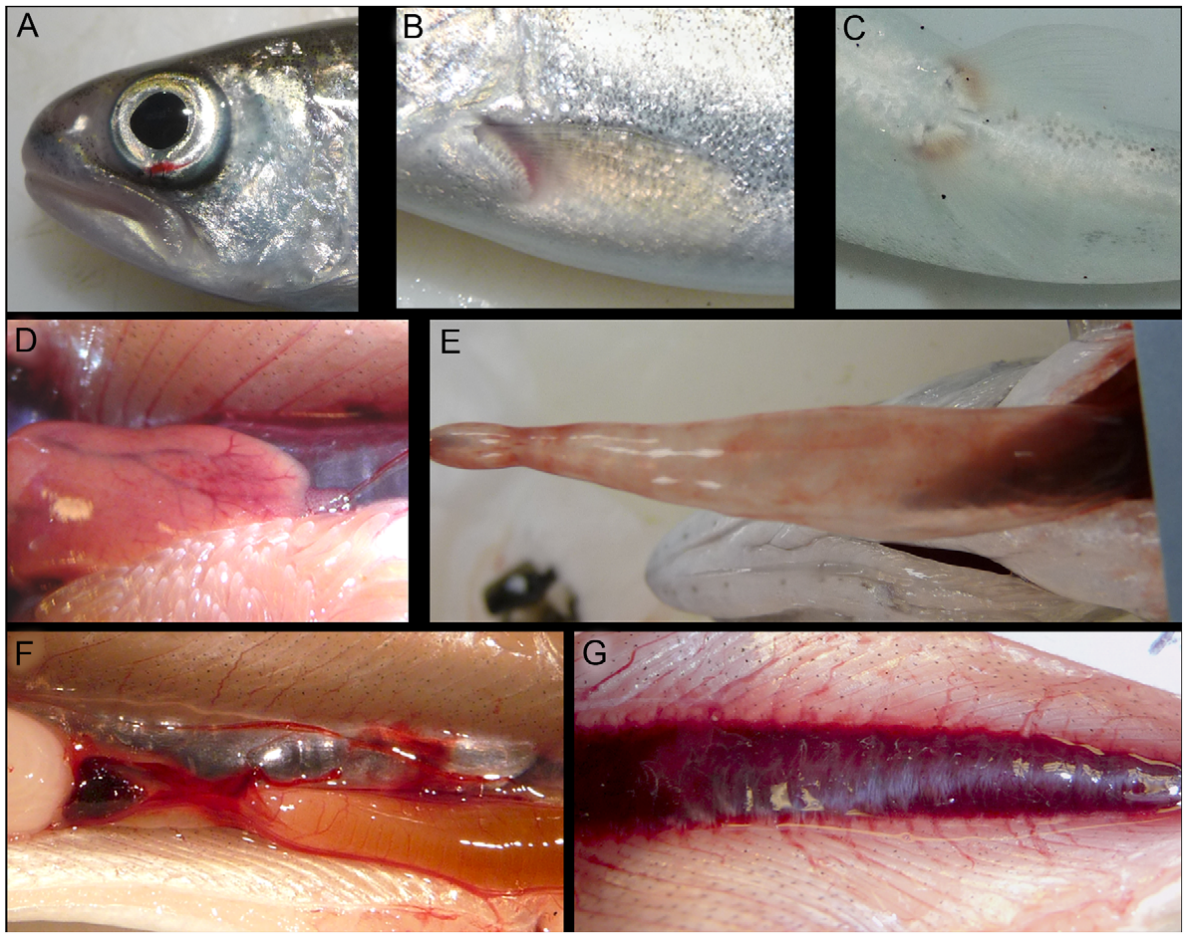
Examples of injuries. Mild injuries are A) eye hemorrhage, B) and C) fin hematoma; Moderate injuries are D) liver hemorrhage and E) bruised swim bladder; Mortal injuries are F) intestinal hemorrhage and G) kidney hemorrhage.

Following exposure in the HICI-FT, and prior to barotrauma examination, fish were euthanized in a buffered solution of tricaine methanesulfonate. Fish were examined for barotrauma injuries both externally and internally then photographed to document injuries (Figure 3).

The investigators were trained to detect and evaluate 62 barotrauma injuries using protocols developed and validated over a number of similar investigations [8], [9], [13], [14], [15], [17], [18]. The investigators use a common methodology to assure uniformity in acquisition and logging of data. Necropsies were conducted using techniques that minimized inadvertent damage to fish organs and tissues.

### Response Variable Derivation - Fish Index of Trauma (FIT) Model

To process the observed injuries, a novel model was developed (called the FIT model) that reflected onset of injury from impulsive sound [8], [18]. For each fish, the presence or absence of external and internal barotrauma injuries were noted in the exposure-response data set. Of the 62 potential injuries, 22 were observed during the study (Table 2). These injuries varied in short- and long-term physiological impacts on fish performance, such as hematoma on fins, broken capillaries, and hemorrhaging organs. Using a medical trauma approach [19], [20], an anatomical scoring system was developed that provided an overall score for fish, regardless of the number of injuries. Injuries were weighted, not by severity or organ, but by known or associated energetic costs of each injury (Woodley and Halvorsen, personal communication; [21], [22]). Many different injury patterns can yield the same score [23]. Weighting allows complex and variable data to be reduced to a single value for each fish.

**Table 2:** List of categorized barotrauma injuries.

Mild	Moderate	Mortal
Hematoma of:	Hemorrhage of:	Dead within 1 hour
Fins	Fins	Hemorrhage of:
Body	Capillaries	Organs
Deflation of:	Hematoma of:	Laceration of:
Swim bladder	Swim bladder	Swim bladder
(not ruptured)	Fat	Organs
	Gonads	
	Muscles	
	Organs	
	GI tract	

Physiological impact of each observed injury was assessed, and then assigned to weighted trauma categories [24], [25]: *Mortal*, *Moderate*, or *Mild* (see Table 2 for details). The *Mortal* trauma category, weighted 5, included injuries that were severe enough to lead to death. The *Moderate* trauma category, weighted 3, included injuries likely to have an adverse impact on fish health but might not lead directly to mortality. Finally, *Mild* trauma category, weighted 1, referred to injuries of minimal to no physiological cost to fish. The weight assignments applied to each of the three trauma categories were based on the assessment of physiological significance that considered the influence of multiple injuries and inspection of data for the occurrence of injury combinations. For example, the occurrence of two injuries categorized as *Moderate* were assessed to have physiological costs similar to one *Mortal* injury. Ultimately, the FIT model provided a weighted score for each fish called the Response Weighted Index (RWI). The RWI is the sum of the presence of each injury multiplied by the trauma weight assigned to each injury type. The formula was:
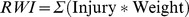
(3)


### Statistical Analysis

The response variable RWI was log transformed before analysis in order to stabilize the variance and linearize the response model. Analysis of covariance (ANCOVA) was performed regressing the transformed RWI against SEL_cum_ and assessing whether the number of impulsive sounds (960 or 1920) had an additional effect on fish response beyond that described by SEL_cum_. Initial analyses were conducted on Treatments 2 through 11 to balance the design because Treatments 2 through 11 were paired. Once a model was selected using a balanced design, Treatment 1 was added to the model.

## Results

**Figure 4:**
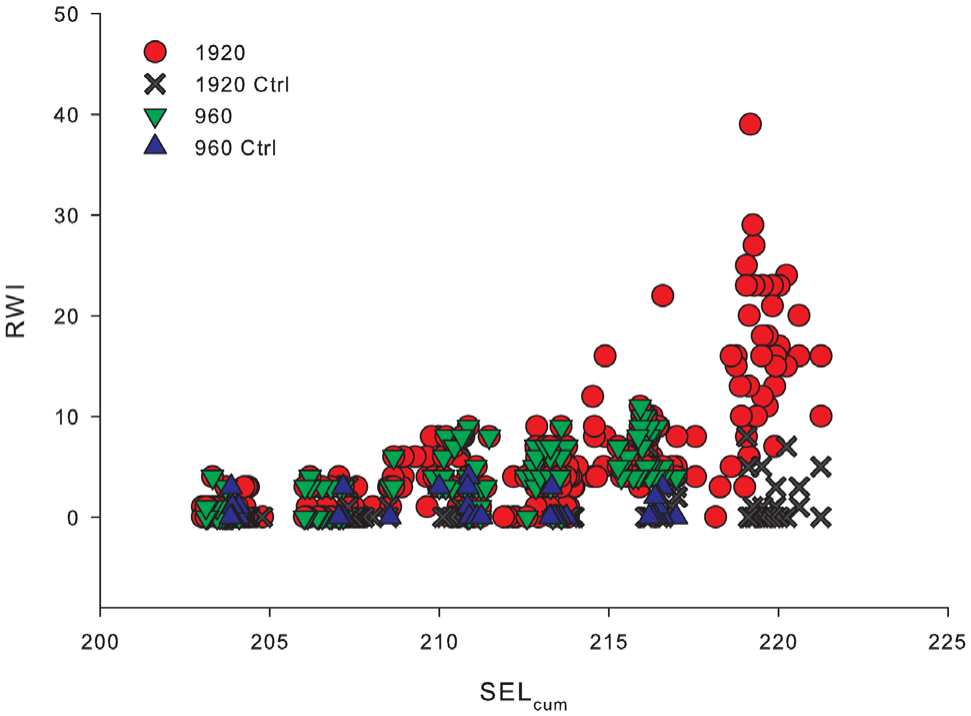
Individual RWI values by SEL_cum_ for 1920 and 960 impulses and controls.

Each Treatment was aimed at a specific SEL_cum_ and SEL_ss_ value. However, small changes of the water compliance in the HICI-FT and small fluctuations in its mechanical operation caused slight differences in the characteristics of individual impulses and consequently the SEL_ss_ and SEL_cum_ values for individual Treatments. This produced a continuum of cumulative energy exposures (±1.5 dB of the target SEL_cum_) rather than specific SEL_ss_ and SEL_cum_ values (Figure 4). Exposure conditions within the HICI-FT chamber, the corresponding exposure metric values, and the average response weighted index (RWI) for the response of test fish are in Table 1.

### Barotrauma Related to SEL_cum_

**Figure 5:**
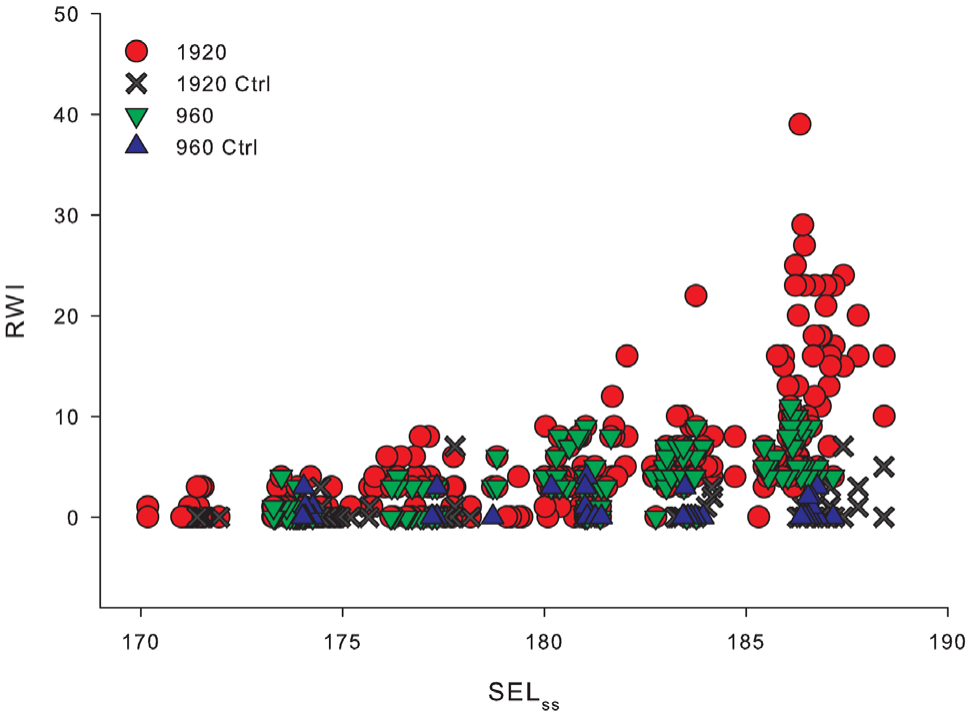
Individual RWI values by SEL_ss_ for 1920 and 960 impulses and controls.

**Figure 6:**
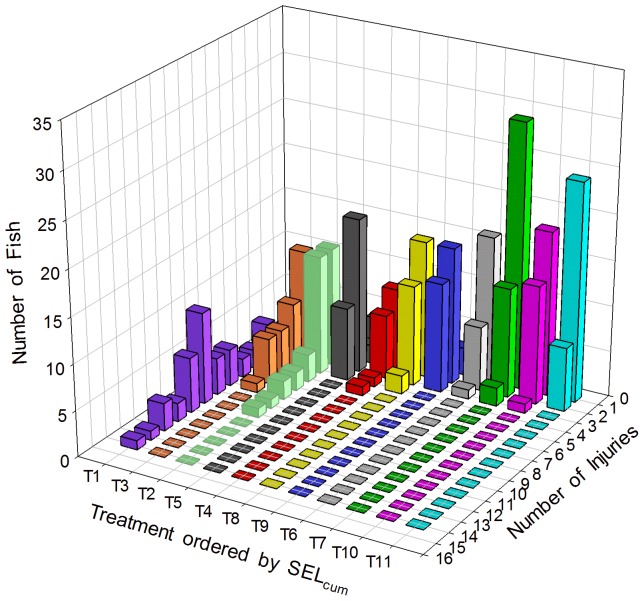
Frequency of barotrauma injury occurrence per fish. The number of test fish (*z*-axis) with number of unweighted-barotrauma injuries (*x*-axis) by each Treatment (*y*-axis) which is in order of SEL_cum_ values (see Table 1). For example, in the most severe exposure (Treatment 1 =  T1, see Table 1 for each Treatment’s metrics), 1 fish had 13 injuries, and 10 fish had 8 injuries. Similarly, for the least severe exposure (T11), 6 fish had 1 injury, and 24 fish had 0 injuries.

Barotrauma injuries ranged from *Mild* to *Mortal*, depending on the amount of energy in the exposure. Mild injuries were those with little if any physiological cost to the fish for example, hematoma on a fin. *Mortal* injuries were those with high physiological cost that could cause death, such as hemorrhaging of the heart. Examples of injuries are shown in Figure 3. The RWI for 1920 and 960 impulsive sounds exposures showed an increase in the extent of physical injury with an increase in SEL_cum_ severity (Figure 4). It was also found that as SEL_ss_ increased, RWI increased exponentially as the number of impulsive sound exposures increased (Figure 5). As RWI increased there were increases in the number of injuries for each exposed fish and physiological impact of those injuries (Figure 6).

**Figure 7:**
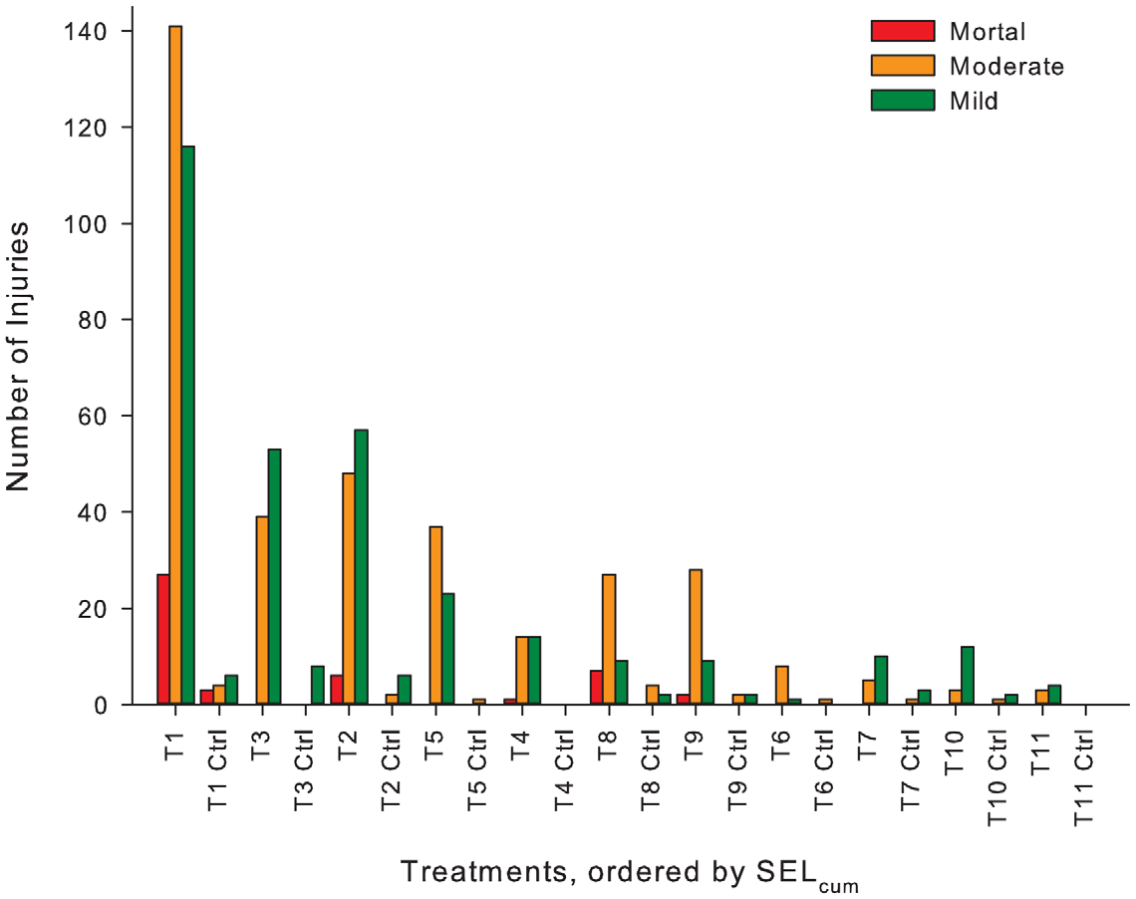
Number of injuries within each injury category. Within each Treatment bin is a representation of the number of injuries for each injury category of *Mortal*, *Moderate*, and *Mild*. The *y*-axis is number of injuries, *x*-axis is each Treatment (exposure and control: ex., T1 =  Treatment 1 Exposure; T1 Ctrl  =  Treatment 1 Control).

There were a few observations of barotrauma injuries in control fish. Injuries that appear in control are reflective of the sensitivity of the FIT model that was used in this study and of the health of the fish. A fish that is expressing a disease and then handled will often show injuries that would not be seen in a healthy fish. Of all documented barotrauma injuries, across all Treatments, 6% of the injuries were in control fish. Within the 6%, 61% of the injuries were *Mild*, 33% were *Moderate*, and 6% were *Mortal*. The three *Mortal* injuries were found in Treatment 1 (Figure 7).

**Figure 8:**
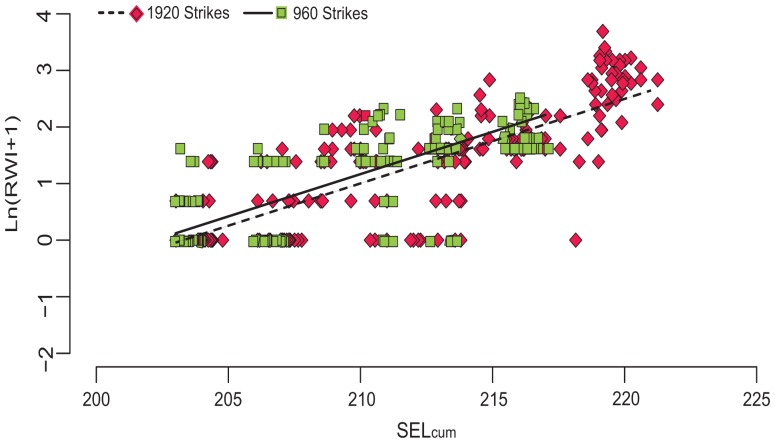
SEL_cum_ vs. ln (RWI+1) for all Treatments. Solid line shows predicted ln(RWI+1) values for 960 strikes and dashed line for 1920 strikes. Green squares denote the 960 strikes and red diamonds denote the 1920 strikes.

Using ANCOVA, it was shown that the regression lines of the log transformed RWI (ln(RWI+1)) versus SEL_cum_ (F_1, 307_ = 0.196, *p* = 0.658) had the same slopes for both 960 and 1920 impulsive sound Treatment sets, but different intercepts (F_1, 308_ = 11.106, *p = *0.001) with the regression line for the 960 impulsive sound exposure lying above that for the 1920 impulsive sound exposure Treatments. A follow on regression analysis where Treatment 1 (SEL_ss_ = 187, SEL_cum_ = 220, number of impulses  = 1920) was added to the 1920 impulsive sound data set did not change the linearity of the model fit to the data or the regression parameters (Figure 8).

The final regression model for the 1920 impulsive sound exposure data set was determined using the data for all Treatments 1–11. The log transformed RWI values showed that fish that experienced 960 impulsive sounds had statistically significant greater RWI values (F_1, 352_ = 6.03; *p* = 0.0145) for all Treatments, than fish exposed to 1920 impulsive sounds at the same values of SEL_cum_ (Figure 8). This is most likely the result of the higher SEL_ss_ for individual impulsive sounds that is required for the 960 Treatments to reach the same SEL_cum_ as 1920 impulsive sound Treatments. The results showed that the severity of fish injury was a function of the energy in SEL_ss_, SEL_cum_, and the number of impulsive sounds.

### Data Integration

**Figure 9:**
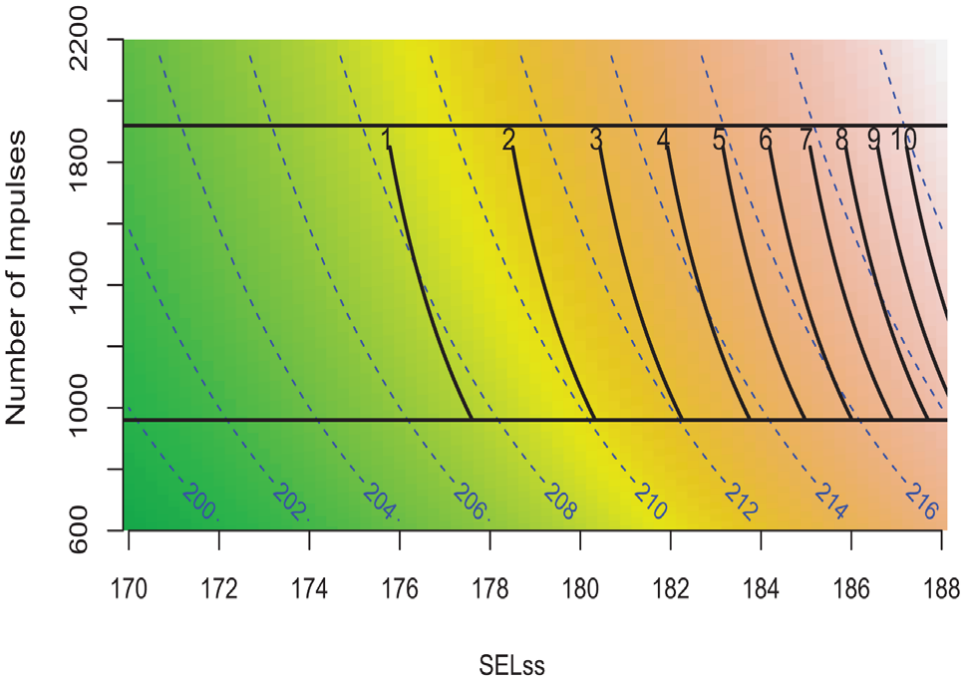
Contour plots of experimental space. The background layer plots the SEL_cum_ contours (blue dashed lines represented by SEL_cum_  =  SEL_ss_ +10log10 (Number of impulses)) by SEL_ss,_ and number of impulses within the Treatment range. The solid black lines labeled 1–10 are a contour plot of the log transformed RWI which illustrates value increases as SEL_ss_ increases; represented by RWI =  exp(−30.050+0.149 * SEL_cum_ –0.000171 * Number of strikes )-1. The upper black horizontal line indicates the 1920 strike-line, and the bottom black horizontal line indicates the 960 strike-line. Together, the plots shows where the RWI contours fall over the SEL_cum_ range and SEL_ss_ range in relation to number of impulses.

The integrated study findings are in Figure 9 and show the relationship between the response of juvenile Chinook salmon, RWI, and the energy in SEL_ss_, SEL_cum_ in an exposure consisting of a number of sequential impulsive sounds. The construction of Figure 9 is one contour plot overlain on a background contour plot. The background contour plot shows the sample space for the study. The background contour plot *x*- and *y*-axes are SEL_ss_ and number of impulsive sounds, respectively, and the *z*-axis is SEL_cum_. The dashed contours show specific SEL_cum_ values and are plotted on the multicolor background that provides additional information about the gradation in SEL_cum_ over the plot surface.

The RWI values (*z*-axis) are represented in Figure 9 by the solid black contour lines. The *x*- and *y*-axes for the RWI contour plot are the same as those for the study sample space (background contour). Note that the top horizontal black line represents 1920 impulses and the lower horizontal black line represents 960 impulses. The curvilinear contours were derived using the results of testing at 1920 and 960 over the range of SEL_cum_ Treatment values and show the RWI values (1–10) that are represented by; RWI  =  exp(−30.050+0.149 * SEL_cum_ –0.000171 * Number of impulses )-1.

## Discussion

### Overview

This is the first laboratory-based study to evaluate the effects of impulsive sounds, under plane-wave acoustic conditions, on neutrally buoyant juvenile fish. The relationship between barotrauma injury to fish and specific sound characteristics, such as number of impulsive sound exposures and sound energy level both SEL_ss_ and SEL_cum_ was systematically explored for onset of injury. The present study demonstrated that the severity of barotrauma, characterized using the FIT model and RWI units, is positively correlated with the energy in each impulsive sound (SEL_ss_), which can be summed over the total number of impulsive sounds generated by the number of pile strikes needed to drive a pile, SEL_cum_ (Figure 8). The highest energy exposures presented in this study, given over 960 and 1920 strikes, caused *Mortal* injuries that resulted in organ hemorrhages that are likely to result in mortality. Lower energy exposures caused fewer barotrauma injuries, and these tended to be injuries found in the *Mild* category (Figure 7), such as fin hematoma, which has minimal physiological effects on the fish.

It is not possible to compare the work here with earlier studies of pile driving sound since those studies used caged fishes under conditions in which the investigators were unable to control the physiological state of the test fish at exposure or any aspects of sound presentation (e.g., number of impulsive sounds, SEL_ss_ or SEL_cum_). In addition, investigators of previous field studies often did not have adequate biological control groups (e.g., [2], [3], [5], [6], [7], [26]). While not clearly stated, the methodologies used in earlier studies suggest that the fish may not have been neutrally buoyant, thereby leaving the validity of the results open to question. It is imperative that future studies examining effects of any impulsive sound be conducted on animals that are determined to be neutrally buoyant, as was done in the present experiment.

### Rejection of the Equal Energy Hypothesis for Impulsive Signals

The “equal energy hypothesis” (EEH) has been suggested as an applicable metric for mitigation of effects of impulsive sound exposure on fish [27], [28]. This hypothesis states that the same type and severity of injury would occur for the same total energy level of exposure (SEL_cum_), regardless of how the total energy was reached (e.g., a large number of low energy impulsive sounds or fewer high energy impulsive sounds) [29]. More recently, studies have shown that this hypothesis is not valid for impulsive sound exposure in mammals [30], [31], [32], and data from the present experiment also rejects the EEH for fish. The data show the statistically significant difference (*p* = 0.0145) between the 1920- and 960-strike regression lines (Figure 8). The difference in SEL_ss_ resulted in a difference in severity of injury despite the equality of SEL_cum_ for study Treatments. Thus, the SEL_cum_ alone is not sufficient to predict the risk of injury to exposed fish. When managing an activity that generates impulsive sound, the SEL_cum_ is an important variable to consider, along with the SEL_ss_ and the number of impulses.

### Impulsive Sound Levels Relative to Injury Consequences

The RWI metric generated by the FIT model allowed for the identification of injury thresholds from impulsive sound exposure, and to define the onset of injury as it relates to impulsive sound [8]. The chance of survival for fishes injured by exposure to impulsive sound depends on the cumulative effect of barotrauma injuries on the physiological function of the fish. The *Mortal* injuries have a clear impact on physiological function such as damage to vital organs. *Moderate* injuries would require considerable opportunity for recovery that, under most circumstances, would be unavailable to the fish (e.g., predator free refuge, ideal flow rates, easily accessed nutrition rich foraging). The *Mild* injuries likely would not affect vital life functions nor swimming performance though physiological costs of healing may still be incurred. The *Mild* injuries singularly or in combination would be unlikely to reduce physiological function or affect the individual’s behavior. Therefore, *Mild* injuries were quantified as below threshold of effects, or injuries that would have only minor physiological or behavioral cost to the fish, although this needs to be tested. A RWI value of 1 or 2 can only occur if a fish has 1 or 2 *Mild*. A RWI value of 3, occurs with one moderate injury or three *Mild* injuries and thus the physiological functioning on some level would be impaired, and consequently fish survival probability starts to decline. The threshold for injury should consider the severity of injury, the category of injury, and the number of incurred injuries (see Figure 7). All these variables are taken into account by the FIT model and the RWI metric.

A RWI value of 2 is suggested to be used to identify the impulsive sound exposure criteria at the threshold of physical injury to juvenile Chinook salmon that, if exceeded, may likely result in physiological function and/or behavioral changes that will impact the survival of the exposed fish. Due to differences among species, life stages, and water quality, this recommendation applies to juvenile Chinook salmon, average length of 103 SL mm and an average wet weight of 11.8 g. A RWI of 2 could be carefully extrapolated to include other fish within the salmonid family of similar size. It is unclear at this time whether other species of fish would show the same injury response to impulsive sound exposure as the juvenile Chinook salmon used in this study.

### Application of RWI

The integrated contour plot (Figure 9) can be used to estimate the exposure conditions corresponding to a particular RWI level of interest or conversely, a RWI can be estimated from a particular set of exposure conditions within the bounds of the data for this study. For example, a RWI of 2 would be achieved for an exposure to 960 impulsive sounds when SEL_ss_ is 180 dB, yielding a SEL_cum_ of 210 dB, and for an exposure to 1920 impulsive sounds when SEL_ss_ is 177 dB yielding a SEL_cum_ value of 210 dB. By plotting the SEL_cum_ and RWI contour plots together onto one graph their relationship to each other as well as their relationship to SEL_ss_ and number of strikes become apparent. While complex, it links a common metric used to manage the exposure of fish to impulsive sound, SEL_cum_, through its constituent parts, SEL_ss_ and number of impulsive sounds, along with the physical injury response variable, RWI.

The most important sound variables to which fish were exposed were the SEL_ss_ and the number of strikes in the case where each pile strike resulted in an impulsive sound with the same energy. These two variables can be used to control activities that generate impulsive sounds, either through management of the energy applied to a pile during each strike or by implementation of mitigating and monitoring actions. This study focused on impulsive sounds and it is reasonable to conclude that these sound level metrics could be extrapolated to other impulsive sounds, such as those generated by seismic exploration.

### Implications of Results with Change in Depth

The experiments described here were performed at absolute pressures equivalent to water surface (1 Atm). However, fish exposed to impulsive sounds in the wild are more likely to occupy greater depths, and could potentially change depth during activity generating impulsive sounds. Thus, the question arises as to the applicability of these results to fish at different depths.

Depth is a variable that may change the barotrauma injuries in fishes from impulsive sounds in deep water. Studies on the effects of rapid decompression on fishes have shown that the magnitude of the ratio of pressure to which fishes are acclimated and the pressure at which fishes are exposed is proportional to the severity of barotrauma injury [13]. If this ratio extends to pile driving and seismic impulsive sounds, it would introduce depth as another variable into the assessment of the effects of these sounds. The result would be a rapid decrease in the severity of exposure and biological response from relatively small increases in depth, given that the static pressure in water increases by about 100 kPa per 10 m of depth. Research is needed to determine if the relationship between acclimation- and exposure-pressures, and if response severity is the same for impulsive sound exposure as it is for rapid decompression.

### Conclusion

The study’s experimental strategy was to determine the relationship between SEL_ss_, SEL_cum_, number of strikes, and response to exposure. The principal result is that estimation of exposure conditions to impulsive sound can be used to manage the risk of physical injury to exposed juvenile Chinook salmon for any selected RWI value. The research results reported here start with a selected level of biological response that protects individuals in an exposed area from injuries that affect performance and/or energetics. The selected biological response level and the results of this study can be used to identify a level of exposure to assure protection of the fishes of concern.

The consequence of these findings is that the severity of injury to fish exposed to impulsive sound cannot be predicted from the SEL_cum_ alone in an exposure consisting of many impulsive events and must consider the energy in the individual impulsive sounds (SEL_ss_) as well the number of impulses that constitute the exposure. The importance of this combination of metrics is made clear for a RWI of 2 as the threshold for onset of injury. A RWI of 2 is reached by an exposure to 960 impulsive sounds when SEL_ss_ is 180 dB, deriving a SEL_cum_ of 210 dB or by an exposure to 1920 impulsive sounds when SEL_ss_ is 177 dB, yielding a SEL_cum_ of 210 dB.
